# A Fab-Selective Immunoglobulin-Binding Domain from Streptococcal Protein G with Improved Half-Life Extension Properties

**DOI:** 10.1371/journal.pone.0139838

**Published:** 2015-10-02

**Authors:** Felix Unverdorben, Meike Hutt, Oliver Seifert, Roland E. Kontermann

**Affiliations:** Institute of Cell Biology and Immunology, University of Stuttgart, Stuttgart, Germany; Vrije Universiteit Brussel, BELGIUM

## Abstract

**Background:**

Half-life extension strategies have gained increasing interest to improve the pharmacokinetic and pharmacodynamic properties of protein therapeutics. Recently, we established an immunoglobulin-binding domain (IgBD) from streptococcal protein G (SpG_C3_) as module for half-life extension. SpG_C3_ is capable of binding to the Fc region as well as the CH1 domain of Fab arms under neutral and acidic conditions.

**Methodology/Principal Findings:**

Using site-directed mutagenesis, we generated a Fab-selective mutant (SpG_C3Fab_) to avoid possible interference with the FcRn-mediated recycling process and improved its affinity for mouse and human IgG by site-directed mutagenesis and phage display selections. In mice, this affinity-improved mutant (SpG_C3Fab_RR) conferred prolonged plasma half-lives compared with SpG_C3Fab_ when fused to small recombinant antibody fragments, such as single-chain Fv (scFv) and bispecific single-chain diabody (scDb). Hence, the SpG_C3Fab_RR domain seems to be a suitable fusion partner for the half-life extension of small recombinant therapeutics.

**Conclusions/Significance:**

The half-life extension properties of SpG_C3_ can be retained by restricting binding to the Fab fragment of serum immunoglobulins and can be improved by increasing binding activity. The modified SpG_C3_ module should be suitable to extend the half-life of therapeutic proteins and, thus to improve therapeutic activity.

## Introduction

With more and more small recombinant protein therapeutics advancing into the clinic, a major task in protein engineering is to overcome the limitation of the rapid clearance from a patient’s bloodstream and to generate efficient and long-lasting pharmaceuticals, e.g. by implementation of half-life extension strategies [1–3]. One approach is to increase the hydrodynamic radius in order to prevent the protein from rapid renal filtration. Measures to circumvent this are for example coupling of hydrophilic polymers such as polyethylene glycol (PEG) and hydroxyethyl starch, and genetic engineering to introduce (additional) glycosylation sites or hydrophilic and flexible polypeptide chains [4–6]. Furthermore, immunoglobulins and also albumin are serum proteins that show the longest circulation time at all and are therefore interesting fusion or interaction partners [7,8]. Their astonishing long circulation time is mediated by the binding, after intracellular uptake, to the neonatal Fc receptor (FcRn) under acidic conditions in a recycling endosome [9–11]. Hence, the proteins are transferred back to the cell surface and upon reaching neutral pH released into the bloodstream, thus being salvaged from lysosomal degradation. Fusion of the Fc part or albumin is known to elongate the half-life of therapeutic proteins, but also transient interactions through IgG- and albumin-binding peptides and protein domains are able to mediate a long circulation time [1,12,13].

Recently, we found that amongst several tested immunoglobulin-binding domains (IgBD) from staphylococcal protein A (SpA) and streptococcal protein G (SpG), the domain C3 of protein G (SpG_C3_) had the best properties in improving the pharmacokinetic profile of a bispecific single-chain diabody (scDb) fusion protein [13,14]. This domain, composed of 56 amino acids, has binding sites for the Fc and the Fab fragment of long-circulating IgG molecules. Its primary binding site is formed by the CH2 and CH3 domain of the Fc part, overlapping with the binding site of the neonatal Fc receptor [15,16]. Distinct from this site, the second binding site is located on the CH1 domain of the Fab arms [17]. The SpG_C3_ domain was able to extend the plasma half-life of the 53 kDa large scDb approximately 18-fold due to binding to serum immunoglobulins after intravenous injection, primarily enhancing the hydrodynamic radius of the complex and thus preventing rapid renal clearance. Of note, the domain was able to bind to IgG also at acidic conditions [14]. Thus, in addition to reduced renal clearance, IgBDs may also be co-recycled via the FcRn as cargo molecule in an acidified endosome.

To investigate the effects of the Fab binding site of the SpG_C3_ domain on half-life extension, we generated a variant of the SpG_C3_ domain (SpG_C3Fab_) with strongly reduced Fc binding activity. This variant was characterized for its binding properties *in vitro* and their capacity to extend the half-life of recombinant antibody molecules. Applying phage display selection of SpG_C3Fab_ libraries, we further identified a variant with improved binding and half-life extension properties.

## Results

### Generation of a Fab-selective SpG-C3

**Fig 1:**
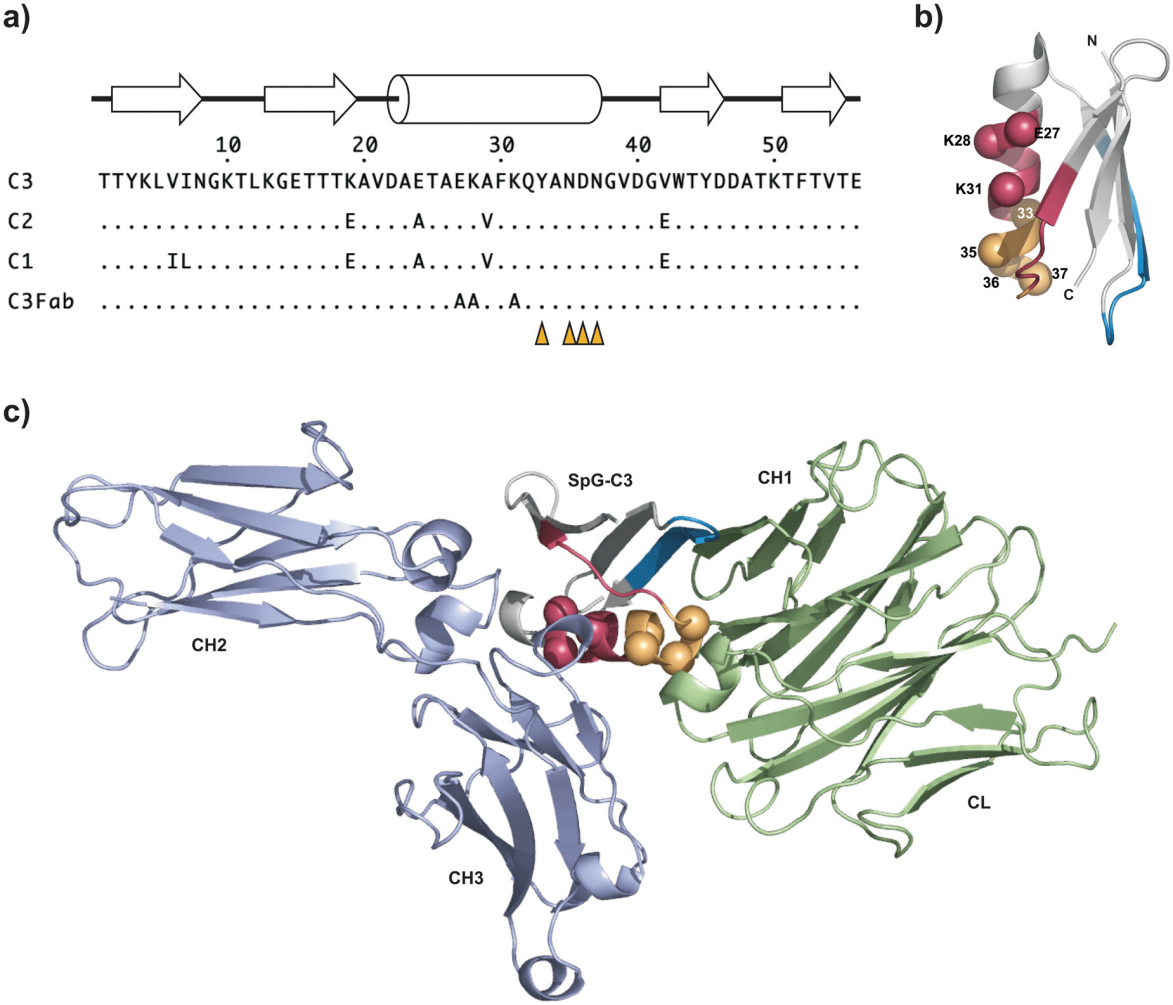
Fab and Fc binding sites in SpG_C3_. a) Alignment of the different SpG IgBDs (C1-C3) as well as SpG_C3Fab_ with mutated Fc binding site. The position of β-strands and the α-helix is indicated [17,19]. Positions randomized in the phage library are indicated by orange triangles (residues 33, 35, 36, and 37). b) Structure of SpG_C3_ (pdb entry 1IGC [17]). The Fc binding site is shown in red/yellow, the major Fab binding site in blue, and the minor Fab binding site in yellow, overlapping with the Fc binding site. Residues mutated to alanines are indicated as red spheres, positions mutated in the phage library are highlighted as orange spheres. c) Superposition of SpGC3 bound to an Fc fragment (pdb entry 1FCC) and a Fab fragment (1IGC) (variable domains are not shown). Residues are marked as in b).

**Fig 2:**
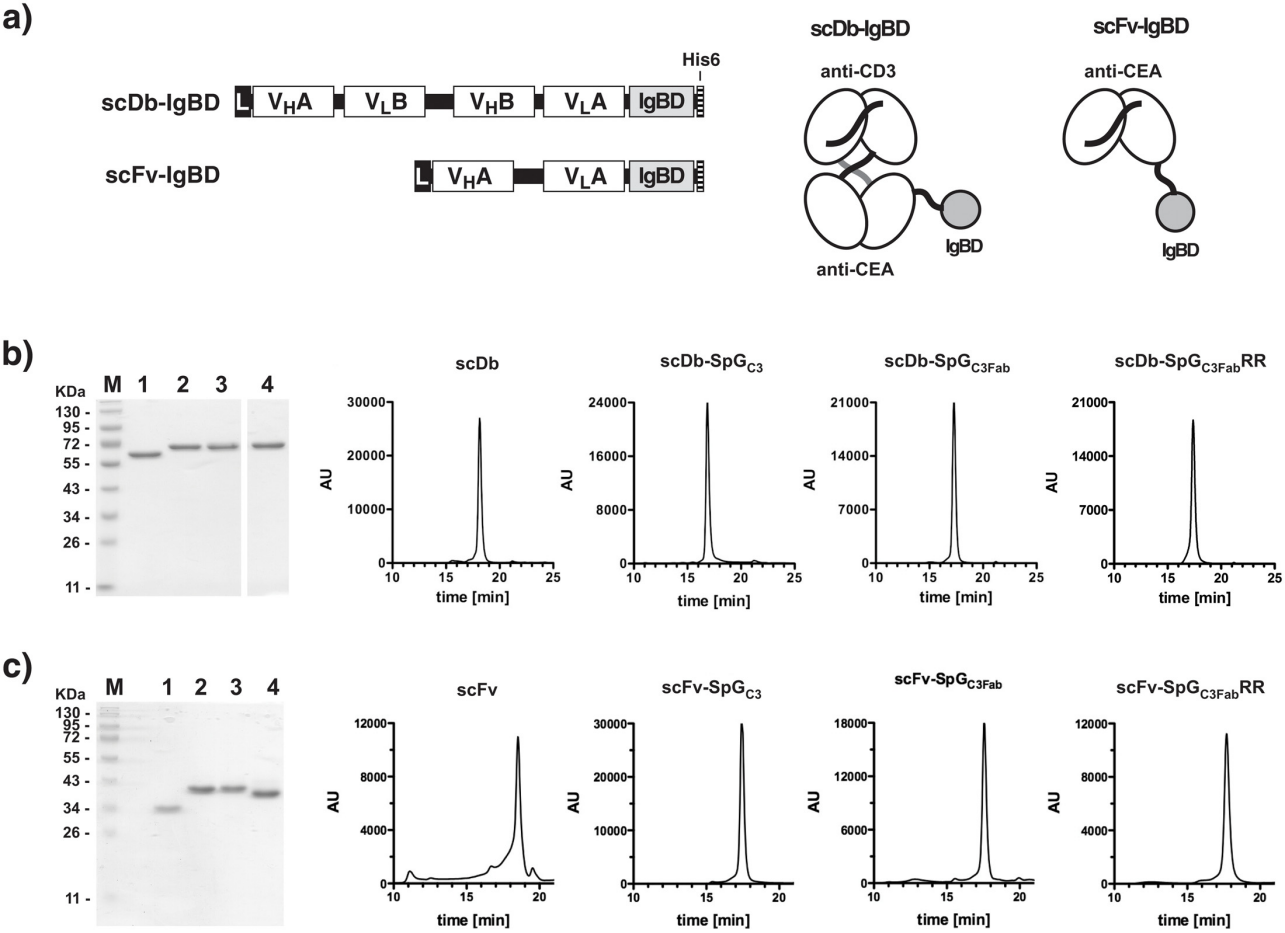
Biochemical characterization of antibody-IgBD fusion proteins. a) Composition of the bispecific scDb-IgBD and the monovalent scFv-IgBD fusion protein. b) SDS-PAGE analysis and HPLC size exclusion chromatography of scDb (1), scDb-SpG_C3_ (2), scDb-SpG_C3Fab_ (3), scDb-SpG_C3Fab_RR analyzed under reducing conditions. c) SDS-PAGE analysis and HPLC size exclusion chromatography of scFv (1), scFv-SpG_C3_ (2), scFv-SpG_C3Fab_ (3), scFv-SpG_C3Fab_RR analyzed under reducing conditions.

The three immunoglobulin-binding domains (IgBD) of Streptococcus protein G (SpG_C1_, SpG_C2_, SpG_C3_) possess two distinct binding sites on IgG molecules. One site is located in the CH1 domain of the Fab fragment and one site in a region formed by the CH2 and CH3 of the Fc fragment. The latter site overlaps with the binding site of the neonatal Fc receptor (FcRn) [15] and could, therefore, potentially interfere with FcRn-mediated recycling. Hence, in a first approach we attempted to eliminate this binding site in SpG_C3_ to restrict binding to the Fab fragment. Three residues in the helical structure of SpG_C3_ (aa Glu27, Lys28, Lys31; numbering as in Fig 1) involved in binding to the Fc region, as deduced from the crystal structure of the homologous domain C2 bound to the Fc region of human IgG [18], were substituted by alanines (SpG_C3Fab_) (Fig 1). SpG_C3_ and SpG_C3Fab_ were fused C-terminally to a 55 kDa bispecific single-chain diabody directed against CEA and CD3 endowed with a hexahistidyl-tag at the very C-terminus. Fusion proteins were produced in stably transfected HEK293T cells and purified from the cell culture supernatant by immobilized metal affinity chromatography. Protein purity was confirmed by SDS-PAGE analysis and by size exclusion chromatography (Fig 2a and 2b). All proteins showed single bands corresponding to their calculated molecular mass of 60 kDa (Table 1) with hydrodynamic radii between 3.0 and 3.2 nm.

**Table 1:** Pharmacokinetic properties (n = 3–6).

Protein	Mol. Mass	S_*r*_	t_1/2_α	t_1/2_β	AUC
	[kDa]	[nm]	[h]	[h]	[%h]
scDb	52.9	2.6	0.4 ± 0.1	1.3 ± 0.2	56 ± 15
scDb-SpG_C2_	59.6	2.9	4.3 ± 0.9	17.3 ± 4.1	1364 ± 332
scDb-SpG_C3_	59.6	3.2	3.8 ± 1.4	24.3 ± 6.8	1879 ± 279
scDb-SpG_C3Fab_	59.6	3.0	3.7 ± 2.7	15.1 ± 4.8	1027 ± 506
scDb-SpG_C3Fab_RR	59.5	3.0	2.1 ± 0.3	47.8 ± 10.3	2228 ± 380
scFv	26.7	2.5	0.2 ± 0.1	0.6 ± 0.2	16 ± 4
scFv-SpG_C3_	33.3	2.9	1.2 ± 0.5	13.1 ± 6.1	426 ± 175
scFv-SpG_C3Fab_	33.2	2.8	1.0 ± 0.1	4.4 ± 1.6	252 ± 43
scFv-SpG_C3Fab_RR	33.2	2.8	0.9 ± 0.1	15.7 ± 9.4	458 ± 229

### Binding Properties of Fab-selective SpG_C3Fab_ fused to a single-chain Diabody

**Fig 3:**
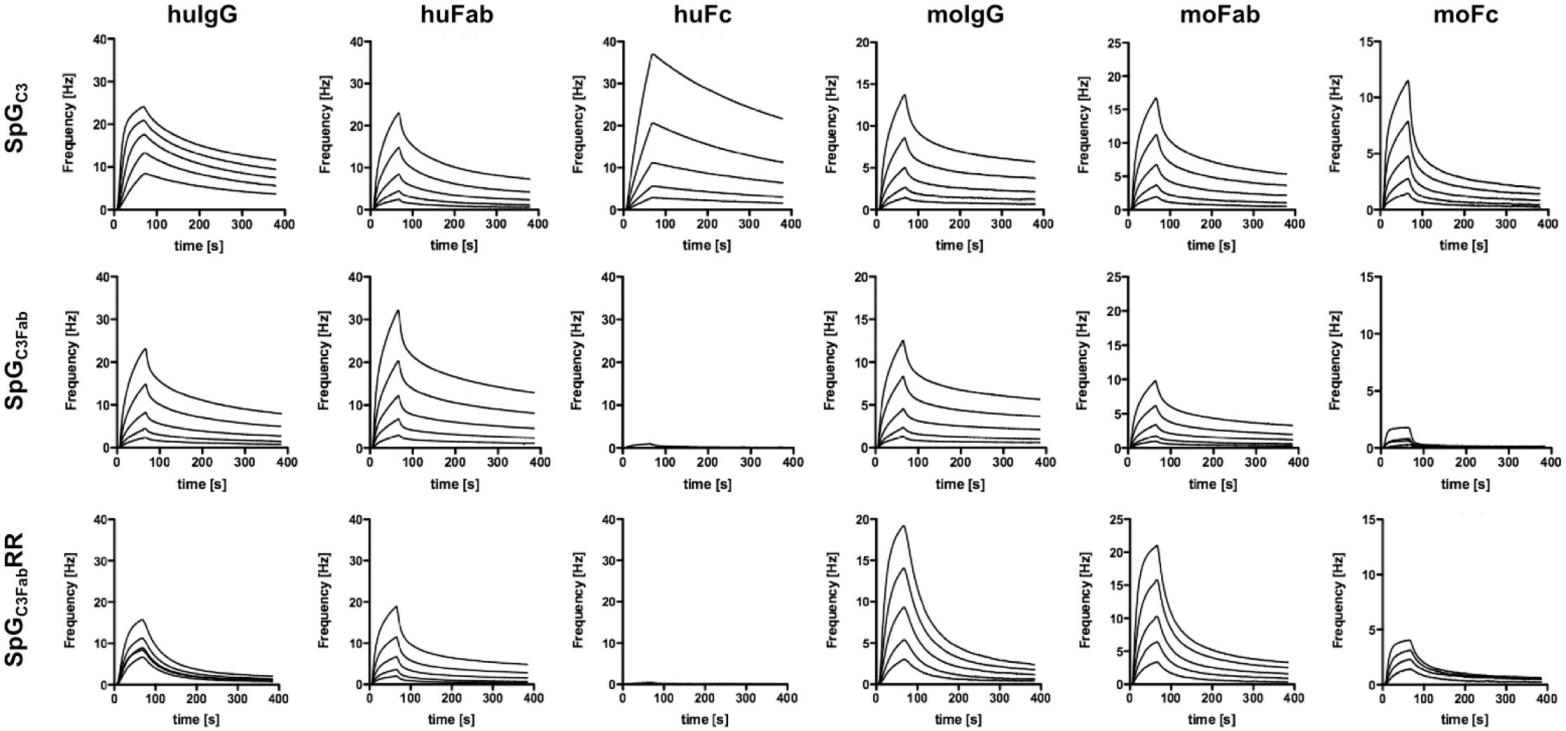
Determination binding kinetics by quartz crystal microbalance measurements. Specificity and affinity of scDb-SpG_C3_, scDb-SpG_C3Fab_, and scDb-SpG_C3Fab_RR for human and mouse serum IgG, Fc Fragments, and Fab fragments were determined by QCM measurements at pH 7.4. Two-fold dilutions were added, starting at a concentration of 1,000 nM.

IgBD-mediated binding of the fusion proteins to immobilized IgG and fragments thereof was investigated by quartz crystal microbalance (Fig 3). Binding curves had to be fitted assuming biphasic kinetics, presumably due to heterogeneity of the immunoglobulin preparations obtained from human and mouse serum, respectively. Unmodified scDb-SpG_C3_ showed strong binding to human and mouse IgG as well as their Fab and Fc fragments, both at neutral pH (pH 7.4) and acidic pH (pH 6) (Table 2). In contrast, for scDb-SpG_C3Fab_ binding was only observed for human and mouse IgG and the Fab fragments, while no or only negligible binding was detected for the Fc fragments at neutral pH (Fig 3). Importantly, compared to scDb-SpG_C3_, the affinity of the SpG_C3Fab_ domain towards the Fab fragment was preserved or even slightly improved at neutral and at acidic pH for both, human and mouse IgG, while a weaker binding of scDb-SpG_C3Fab_ to full-length IgG was observed at neutral pH (Table 2). Interestingly, at acidic pH SpG_C3Fab_ was able to bind to the Fc fragment to a certain extent, especially mouse Fc. These experiments established that the introduced alanine substitutions at positions Glu27, Lys28, and Lys31 specifically deleted the Fc binding site in scDb-SpG_C3Fab_, at least at neutral pH, without affecting Fab binding.

**Table 2:** Affinity determination by QCM (K_D_ values in nM).

Protein		human						mouse					
		IgG		Fab		Fc		IgG		Fab		Fc	
	pH	K_D1_	K_D2_	K_D1_	K_D2_	K_D1_	K_D2_	K_D1_	K_D2_	K_D1_	K_D2_	K_D1_	K_D2_
scDb-SpG_C3_	7.4	3	38	159	663	6	17	33	593	118	1100	79	926
	6	2	33	95	667	1	28	27	406	130	874	53	858
scDb-SpG_C3Fab_	7.4	120	983	88	969	-	-	61	1030	96	1620	-	-
	6	169	1520	83	753	767	896	54	822	93	826	38	414
scDb-SpG_C3Fab_RR	7.4	29	385	167	1040	-	-	55	388	62	413	-	-
	6	49	191	43	461	39	227	34	296	19	255	40	289

**Fig 4:**
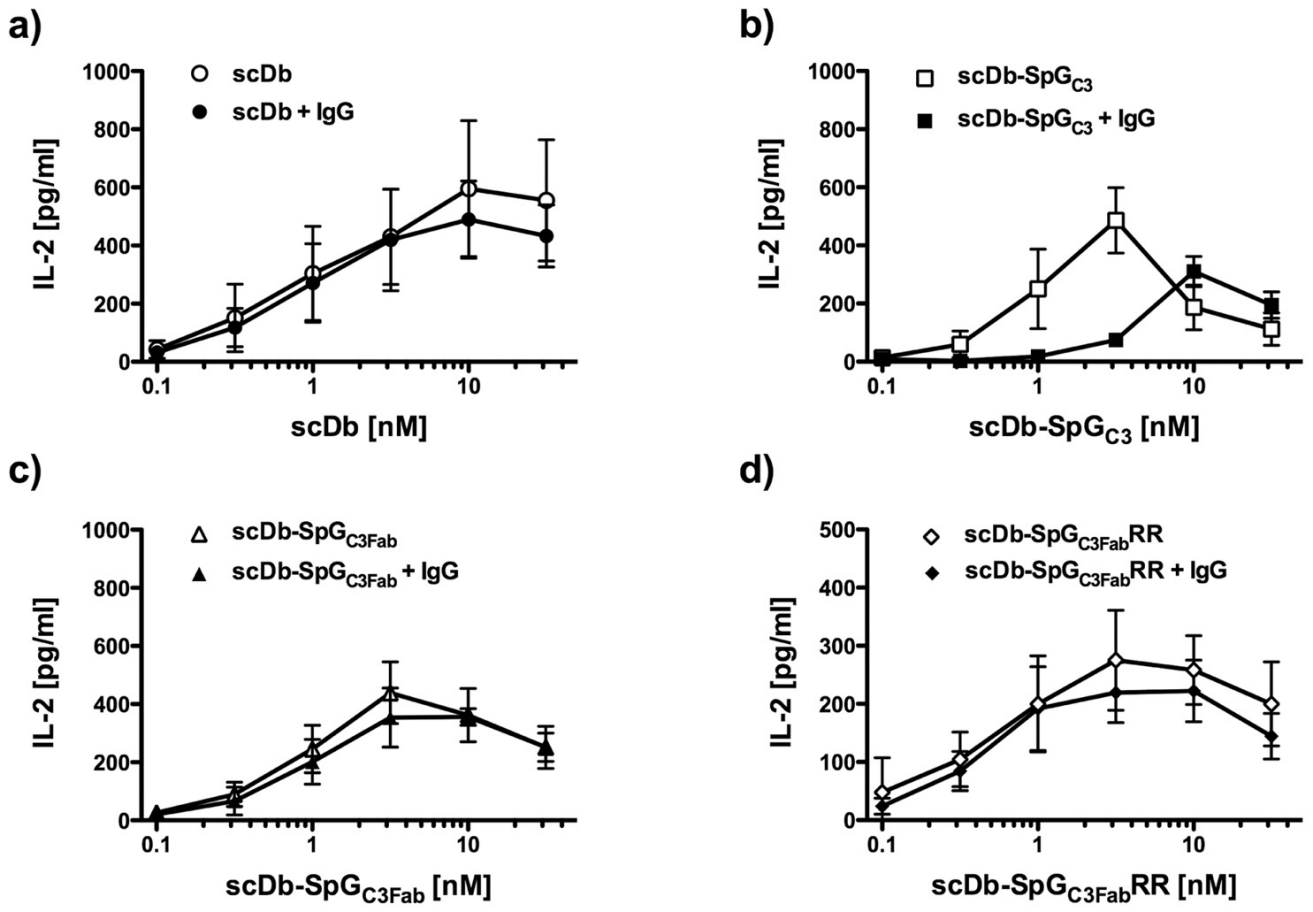
Effector cell retargeting and activation by bispecific scDb fusion proteins determined by IL–2 release. CEA positive target cells (LS174T) were incubated with scDb-SpG_C3_ variants together with or without 100 μg/ml human IgG 1 h prior to addition of PBMCs. Cell free supernatant was analyzed after 24 h for released IL-2 by ELISA.

Next, a functional assay was performed to analyze the effector cell recruiting and activating activity of the scDb moiety capable of crosslinking tumor cells and T-cells, thereby leading to the activation of the effector T-cells. Adherent CEA-expressing LS174T cells were incubated with human PBMCs as effector cells together with the scDb fusion protein and activation of the effector T cells was determined by quantification of induced IL–2 release. All scDb-fusion proteins were able to activate the IL–2 secretion of PBMCs in a dose-dependent manner, reaching the maximal stimulating concentration at 3 to 10 nM in accordance with previous findings [12] (Fig 4a–4c). In the absence of human IgG, all scDb fusion proteins behaved as the scDb control. However, in the presence of IgG, scDb-SpG_C3_ exhibited strongly reduced activation levels. In contrast, scDb-SpG_C3Fab_-mediated IL–2 release was not affected in the presence of IgG with levels of IL-2 that were similar to the scDb control. Binding of scDb-SpG_C3_ and scDb-SpG_C3Fab_ fusion proteins to CEA-expressing LS174T cells and isolated PBMCs analyzed by flow cytometry was not or only marginally affected in the presence of IgG, indicating that the reduced activation of effector cells by scDb-SpG_C3_ is not caused by reduced target and effector cell binding (data not shown).

### Pharmacokinetics of Fab-selective SpG_C3Fab_ fused to a single-chain Diabody

**Fig 5:**
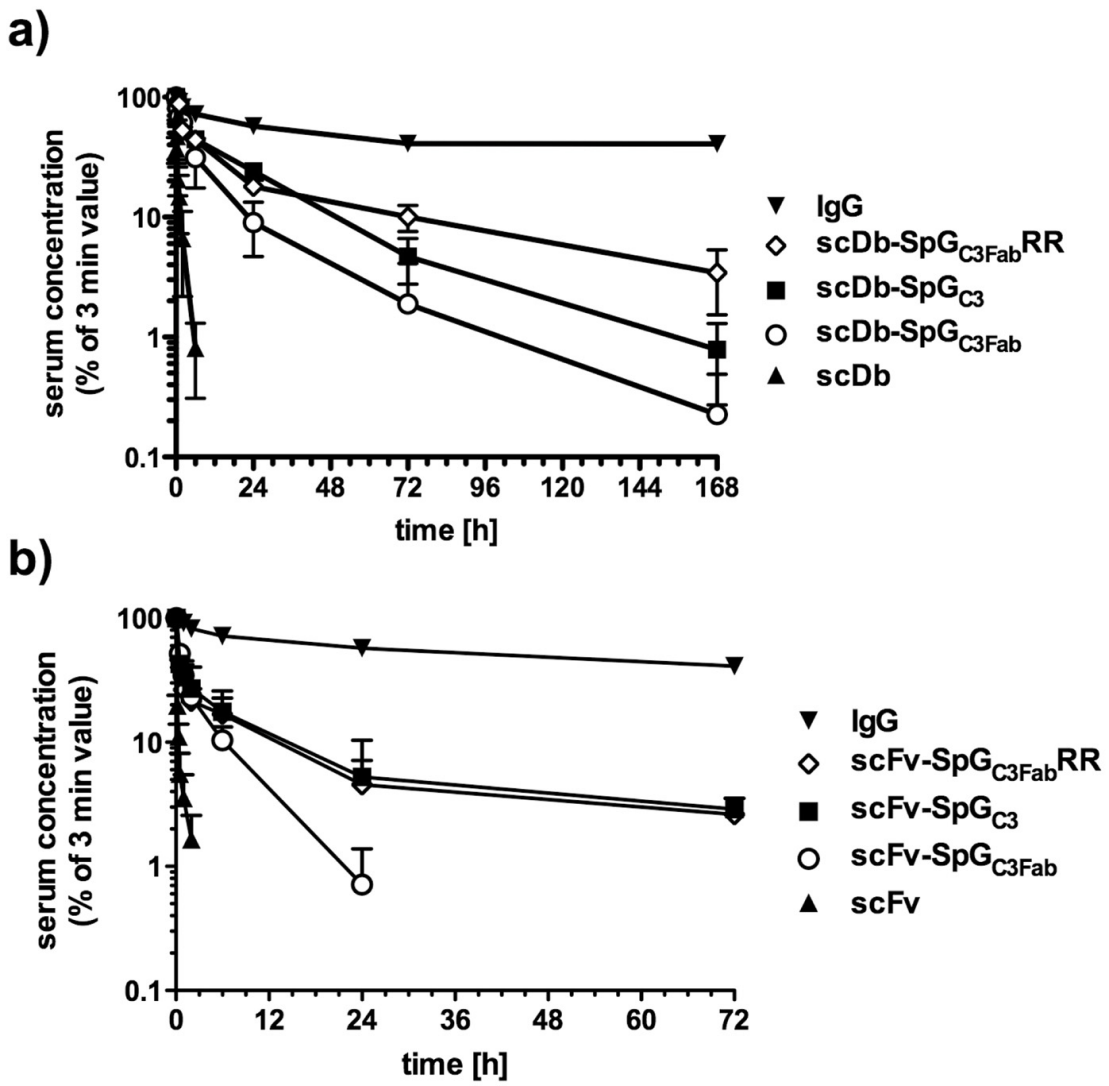
Pharmacokinetics of the fusion proteins in CD1 mice. a) scDb fusion proteins. b) scFv fusion proteins. 25 μg of scDb-SpG_C3_ (a) or scFv-SpG_C3_ (b) variants was injected intravenously into female CD1 mice. Serum concentrations were determined by ELISA against CEA and data were normalized considering the maximal concentration at the first time point (3 min).

The pharmacokinetic properties of scDb-SpG_C3_ and scDb-SpG_C3Fab_ were investigated in female CD1 mice after intravenous injection of the fusion proteins. The serum concentration was determined by ELISA to detect CEA-binding molecules. The unmodified scDb showed rapid clearance from the bloodstream with a terminal half-life of 1.3 h (Table 1, Fig 5). Compared to the unmodified scDb, the scDb-SpG_C3Fab_ fusion protein exhibited a strongly augmented terminal half-life (15.1 h), which was also reflected by a strongly increased AUC (Table 1). However, scDb-SpG_C3Fab_ did not reach the half-life (24.3 h) of scDb-SpG_C3_.

### Affinity maturation of SpG_C3Fab_

The reduced half-life of scDb-SpG_C3Fab_ compared to scDb-SpG_C3_ might be caused by a reduced binding to serum IgG (see Table 2). In order to improve the binding to the CH1 domain of the IgG Fab arms, a phage display library of mutated SpG_C3Fab_ variants was generated. By site-directed mutagenesis, positions 33, 35, 36 and 37, described to be involved in Fab binding [17], were randomized (Fig 1a). After 3 rounds of selection against biotinylated soluble mouse Fab fragments, a polyclonal phage ELISA revealed an enhanced binding of the enriched phage to human and mouse IgG and Fab fragments (data not shown). One clone with two mutations at positions 35 and 36 (N35R, D36R) and wild-type residues at positions 33 and 37 (SpG_C3Fab_RR) showed the best binding properties and was subjected to further analysis as scDb-SpG_C3Fab_RR fusion protein. QCM measurements demonstrated an increased binding to mouse Fab fragment, especially under acidic pH reflected also by a slightly improved affinity towards mouse IgG (Table 2). Similarly, the affinity for human IgG was increased, which was also seen for human Fab fragments at acidic pH, although only marginal differences were determined for binding to human Fab fragments at neutral pH. SpG_C3Fab_RR did not bind to human and mouse Fc fragments at neutral pH, but retained binding to human and mouse Fc fragments at acidic pH, as observed for SpG_C3Fab_. Similar to scDb-SpG_C3Fab_, scDb-SpG_C3Fab_RR showed strong activation of PBMCs in the recruitment experiment, and this activity was not affected in the presence of excess amounts of IgG (Fig 4d).

### Pharmacokinetics of affinity matured SpG_C3Fab_ fused to single-chain Diabody and an scFv

Finally, we determined the pharmacokinetic properties of scDb-SpG_C3Fab_RR in CD1 mice. Here, we included also an anti-CEA scFv with only half the size of the scDb as fusion partner for the various SpG_C3_ variants. In addition, we tested also SpG_C2_, which differs at 4 positions from SpG_C3_, fused to scDb. This fusion protein, scDb-SpG_C2_, exhibited a somewhat lower terminal half-life (17.3 h) compared to scDb-SpG_C3_. For scDb-SpG_C3Fab_RR, we observed a strongly extended terminal half-life (47.8 h) compared with scDb-SpG_C3Fab_, but also compared with scDb-SpG_C3_ (Fig 5, Table 1). Similarly, fusion of SpG_C3Fab_RR to scFv (scFv-SpG_C3Fab_RR) resulted in an increased terminal half-life compared with scFv-SpG_C3Fab_ (15.7 h vs 4.4 h). The terminal half-life of scFv-SpG_C3Fab_RR was similar to that of scFv-SpG_C3_ (13.1 h). The increased terminal half-life was always accompanied with an enhanced AUC value. Again the SpG_C3Fab_RR variant showed the strongest improvements for both fusion partners with almost 40-fold increased AUC compared to the scDb and 28-fold for the scFv.

## Discussion

Immunoglobulins of the IgG class and albumin exhibit exceptionally long serum half-lives and have therefore gained increasing interest for half-life extension strategies [1]. For example, fusion of moieties with affinity for IgG or albumin to therapeutic proteins allows binding to the serum protein in vivo resulting in an extended half-life. While various albumin-binding moieties have been extensively studied for half-life extension, little efforts have been made to use immunoglobulin-binding moieties for this purpose [13,14,19–23]. Several factors might influence the capacity of these immunoglobulin-binding moieties to extend the serum half-life of fusion partners [14]. Amongst those are the selectivity for long-circulating antibody classes and subclasses (especially IgG1, IgG2, IgG4) [24,25], the stability of the complexes in the blood stream (i.e. at neutral pH) and after internalization and during endosomal recycling (i.e. at acidic pH), and the interference with FcRn binding under acidic conditions. Furthermore, the size of the IgBD fusion protein itself might affect the pharmacokinetic properties.

The SpG_C3_ domain can bind to the Fc and Fab fragments of immunoglobulins reflecting the binding specificities of protein G [26], with a preference for IgG [27]. In a previous study, we demonstrated already that SpG_C3_ binds stronger to the Fc fragment than to the Fab fragment [14], which was confirmed here using Fc and Fab fragments from human and mouse serum IgG. By mutating three key residues of the Fc binding site of SpG_C3_ to alanines we were able to generate variants with selectivity for Fab fragments. However, the half-life of an scDb-SpG_C3Fab_ fusion protein was markedly reduced compared to the corresponding SpG_C3_ fusion protein, which was also seen for an scFv-SpG_C3Fab_ fusion protein. Presumably, this is caused by a reduced affinity for serum IgG of SpG_C3Fab_ fusion protein. For that reason, we increased the affinity towards the whole IgG and in particular the Fab fragment by phage display, resulting in the SpG_C3Fab_RR domain.

Using this affinity-improved domain, we were able to increase the terminal half-life of the scDb fusion protein around 40-fold. Of note, the half-life of scDb-SpG_C3Fab_RR (47.8 h) is much longer compared to an IgG that was tested in FcRn knockout mice (19 h compared with 95 h in wild-type mice), indicating a contribution of FcRn-mediated recycling [28]. Mechanistically, it is essential for the IgBDs to retain the binding affinity towards the IgG molecule and especially the Fab fragment under neutral and acidic conditions to avoid dissociation of the bound IgBD fusion protein from the recycling IgG molecule. Both, SpG_C3Fab_ and SpG_C3Fab_RR fulfill this requirement, suggesting that the complexes with the Fab arms are stable also during the endosomal recycling process. The SpG_C3Fab_RR exhibits a better affinity towards the Fab fragment than the SpG_C3Fab_ domain, which should stabilize the complex in the neutral blood as well as during the recycling in the FcRn. It was already described that the SpG_C3_ domain changes its affinity towards the Fc part depending on the pH, probably due to the protonation of histidine residues in the Fc part that are in close proximity to the bound SpG_C3_ domain [18,29,30]. Our data suggest that despite SpGC3FabRR having an increased affinity for the Fc-domain at pH 6 compared to SpGC3Fab, which could inhibit IgG recycling by the FcRn, the half-life of SpGC3FabRR is considerably longer than SpGC3Fab, presumably due to its higher affinity binding to Fab.

Our studies also showed that the size of the IgBD fusion proteins influences the pharmacokinetic properties. Compared with scDb-IgBD fusion proteins, the scFv-IgBD fusion proteins with only half the size exhibited a reduced half-life and AUC. Dissociation of the bound fusion proteins from immunoglobulins in serum and subsequent renal clearance might be responsible. Further affinity maturation of SpG_C3Fab_RR might therefore be beneficial to further extend the half-lives of small size molecules.

SpG_C3_ when fused to the bispecific scDb, designed to retarget effector T cells to tumor cells, reduced the effector cell stimulating activities mediated by binding to CD3 in the presence of IgG. Of note, SpG_C3Fab_ and SpG_C3Fab_RR did not show this reduction. Similar findings have previously been observed for various other half-life extension strategies, e.g. PEGylation and fusion to an albumin-binding domain [5,12]. Our findings indicate that in this application, where effector cells have to come in close contact with the target cells, differences in binding activity of the IgBD have strong effects on the T-cell retargeting and triggering activity. Thus, SpG_C3Fab_ and SpG_C3Fab_RR might be especially useful for the half-life extension of small bispecific T cell engagers [31,32].

In summary, we were able to generate new immunoglobulin-binding domains that bind specifically to the Fab fragment of IgG molecules. The reduced half-life extension properties of SpG_C3Fab_ were compensated by mutations leading to improved affinity. Hence, the SpG_C3Fab_RR domain seems to be a suitable fusion partner for the half-life extension of small recombinant therapeutics.

## Materials and Methods

### Materials

Horseradish peroxidase-conjugated (HRP) anti-His-tag antibody was purchased from Santa Cruz Biotechnology (Santa Cruz, CA, USA). Carcinoembryonic antigen (CEA) was obtained from Antikörper-online.de (Aachen, Germany). Human and mouse serum IgG were purchased from Sigma, Fab and Fc fragments from Emelca Bioscience (Breda, Netherlands). The human colon adenocarcinoma cell line LS174T was purchased from ECACC (Wiltshire, UK) and cultured in RPMI, 5% fetal bovine serum (FBS), 2 mM glutamine (Invitrogen, Karlsruhe, Germany). Buffy coats from anonymous healthy human donors were obtained from blood donation center of the Klinikum Stuttgart (Stuttgart, Germany; http://www.klinikum-stuttgart.de/). The DuoSet IL–2 ELISA Development System kit was purchased from R&D Systems (Nordenstadt, Germany). CD1 mice were purchased from Charles River (Sulzfeld, Germany).

### Cloning and production

The DNA encoding the SpG_C3Fab_ sequence, including the His-tag, was purchased from Geneart (Regensburg, Germany) with a NotI restriction site at the 5’ end and an EcoRI site at the 3’ end to exchange the SpG_C3_ domain from the previously used vector pSecTagA-scDbCEACD3-SpG_C3_ or pSecTagA-scFvCEA-SpG_C3_ [14]. For phage display, degenerate primers were used for PCR with a SalI restriction site at the 5’ end and a NotI site at the 3’ end. NNK triplets were used at the sites that encode for amino acids binding to the CH1 domain (forward primer: 5'-TCG AGT CGA CGC CGA AAC AGC CGC CGC TGC CTT TGC CCA GNN KGC CNN KNN KNN KGG CGT GGA CGG CGT GTG GAC C–3', reverse primer: 5'-ATT TGC GGC CGC CTC GGT CAC GGT GAA GGT–3'). The digested PCR product was ligated into pHEN3-SpG_C3Fab_ΔN (SalI), a modified version of SpG_C3Fab_ with a stop codon at the 5' region generated via cloning into the phagemid vector pHEN3 (based on pHENIX [33]), with an additional His-tag via SalI and NotI. The insert was created by PCR (Forward primer: 5'-TCGAGTCGACAGTACGCCAACGATAATGGCG–3' and reverse primer from above). The ligated library was transformed into *E*.*coli* TG1 by electroporation. The SpG_C3Fab_RR domain was amplified by PCR from the pHEN3 vector and then cloned into pSecTagA-scDb-SpG_C3Fab_ with NotI and BamHI (Forward primer: 5'-ATT TGC GGC CGC AGG CGG ATC TGG CGG AAC AAC ATA CAA GCT CGT G–3', reverse primer: 5'-GAT CGG ATC CGC CCT CGG TCA CGG TGA AGG T–3'). All pSecTag plasmids were stably transfected into HEK293T cells and the resulting fusion proteins were purified from the supernatant by immobilized metal affinity chromatography as described previously [19].

### Phage display

Purified murine Fab fragment was biotinylated with EZ-link™Sulfo-NHS-SS-Biotin from Thermo Scientific (Rockford, USA) according to manufacturer’s protocol. Phages were rescued from library stock or preselected rounds with VSC-M13 helper phages. In the presence of 1% bovine serum albumin (BSA) as blocking reagent in PBS, the phages were incubated with biotinylated Fab fragment (100 nM to 1 nM over 3 rounds) for 1 h at room temperature and then added to streptavidin-coupled M-280 Dynabeads (Thermo Scientific, Rockford, USA) for 15 minutes to allow the complex to bind to the beads. A magnetic separator was used to separate the beads. After washing 4 times with PBST (PBS, 0.1% Tween 20) and twice with PBS, bound phages were eluted with 10 mM dithiothreitol for 5 min and subsequently inoculated to log-phase *E*. *coli* TG1 for further selection rounds. Starting from the second round, washing was performed at pH 6.0.

### Size exclusion chromatography

Purity and Stokes radii of the recombinant fusion proteins were determined by HPLC size exclusion chromatography with a Yarra SEC–2000 column (Phenomenex, Aschaffenburg, Germany). 20 μl of proteins (0.5 mg/ml) were injected at a flow rate of 0.5 ml/min. The following standard proteins were used to calculate the apparent molecular mass and hydrodynamic radius: thyroglobulin (669 kDa), apoferritin (443 kDa), alcohol dehydrogenase (150 kDa), BSA (67 kDa), carbonic anhydrase (29 kDa) and FLAG peptide (1 kDa).

### ELISA

CEA (3 μg/ml) or immunoglobulins (1 μg/ml) were immobilized on ELISA plates over night at 4°C and remaining binding sites were blocked with 2% (w/v) non-fat dry milk/phosphate buffered saline (MPBS). Purified recombinant proteins, serum samples or phage display supernatants were diluted in MPBS, titrated in duplicates and incubated for 1 h at room temperature. Detection was performed with HRP conjugated anti-His-tag antibody or anti-M13 antibody for the phage ELISA using 100 μl 3,3’,5,5’-tetramethylbenzidine (TMB) substrate (0.1 mg/ml TMB, 100 mM sodium acetate buffer pH 6.0, 0.006% H_2_O_2_). The reaction was stopped with 50 μl of 1 M H_2_SO_4_ and absorbance was measured at 450 nm. Data were fitted with GraphPrism software (La Jolla, CA, USA) from three independent binding curves. From these three individual EC_50_ values the mean and standard deviation were calculated.

### Flow cytometry

Binding to CEA-expressing LS174T cells and IL-2-stimulated T cells from peripheral blood mononuclear cells (PBMCs) was determined by flow cytometry [34]. 2.5 x 10^5^ cells were incubated with dilution series of antibodies for 2 h at 4°C. Cells were then washed with PBS, 2% FBS, 0.02% NaN_3_ (PBA) and bound antibodies were detected with PE-conjugated mouse anti-His-tag antibody.

### Interleukin-2 release assay

Peripheral blood mononuclear cells (PBMCs) from healthy donors were isolated from buffy coat as described before [19]. 10^5^ LS174T cells/100 μl/well were seeded in 96-well plates. The next day, supernatant was removed and 150 μl of recombinant antibody was added. After 1 h preincubation at 37°C, 2 x 10^5^ PBMC/50 μl/well were added. PBMCs had been thawed the day before and seeded on a culture dish. Only cells that remained in suspension were used for the assay. After addition of PBMCs, the 96-well plate was incubated for 24 h at 37°C, 5% CO_2_. All incubations were performed in RPMI supplemented with 10% FBS. Plates were centrifuged and cell-free supernatant was collected. Concentration of human interleukin (IL)-2 in the supernatant was determined using the DuoSet IL–2 ELISA kit (R&D Systems) following the manufacturer’s protocol.

### Affinity determination

Affinities of IgBD fusion proteins for human and mouse serum IgG as well as Fab and Fc fragments thereof at neutral or acidic pH were determined by quartz crystal microbalance (QCM) measurements (A200 C-Fast system; Attana, Stockholm, Sweden). The ligands were chemically immobilized on a low nonspecific binding carboxyl sensor chip according to the manufacturer’s protocol at a density resulting in a signal increase of 70–90 Hz. Binding experiments were performed in PBST (0.1% Tween 20), pH 7.4 or pH 6.0, at a flow rate of 25 μl/min. The chip was regenerated twice with 10 mM glycine-HCl, pH 3.0 for 30 seconds. Before each measurement, a base line was measured which was subtracted from the binding curve. Proteins were injected in a random order in two-fold dilutions, starting at a concentration of 1 μM. Data were analyzed with the Attana evaluation software (Version 3.3.4) and TraceDrawer 1.6, using a one-to-two binding model to determine the binding parameters.

### Pharmacokinetics

Animal care and all experiments performed were in accordance with federal and european guidelines and have been approved by Regierungspräsidium Stuttgart. Female CD1 mice (6–12 weeks, 25–35 g) received an intravenous injection of 25 μg of the IgBD fusion proteins in a total volume of 150 μl. In time intervals of 3 min, 30 min, 1 h, 2 h, 6 h, 1 day, 3 days, and 7 days, blood samples (50 μl) were taken from the tail and incubated on ice. Clotted blood was centrifuged at 13,000 g for 30 min at 4°C, and serum samples were stored at -20°C. Serum concentrations of CEA-binding recombinant antibodies were determined by ELISA (as described above). For comparison, the first value (3 min) was set to 100%. Half-lives (t_1⁄2_α, t_1⁄2_β) and area-under-the-curve (AUC) were calculated with Excel. Terminal half-lives (t_1⁄2_β) were calculated using the last three or four serum concentrations shown in Fig 5. For statistics, One-way-Anova was applied with Tukey’s post-test. Results are shown as mean value ± standard deviation.

## References

[1] Kontermann RE (2011) Strategies for extended serum half-life of protein therapeutics. Curr Opin Biotechnol 22: 868–876. 10.1016/j.copbio.2011.06.012 21862310

[2] Constantinou A, Chen C, Deonarain MP (2010) Modulating the pharmacokinetics of therapeutic antibodies. Biotechnol Lett 32: 609–622. 10.1007/s10529-010-0214-z 20131077

[3] Szlachcic A, Zakrzewska M, Otlewski J (2011) Longer action means better drug: tuning up protein therapeutics. Biotechnol Adv 29: 436–441. 10.1016/j.biotechadv.2011.03.005 21443940

[4] Harris JM, Chess RB (2003) Effect of pegylation on pharmaceuticals. Nat Rev Drug Discov 2: 214–221. 12612647 10.1038/nrd1033

[5] Stork R, Zettlitz KA, Müller D, Rether M, Hanisch FG, Kontermann RE (2008) N-glycosylation as novel strategy to improve pharmacokinetic properties of bispecific single-chain diabodies. J Biol Chem 283: 7804–7812. 10.1074/jbc.M709179200 18211902

[6] Schellenberger V, Wang CW, Geething NC, Spink BJ, Campbell A, To W, et al (2009) A recombinant polypeptide extends the in vivo half-life of peptides and proteins in a tunable manner. Nat Biotechnol 27: 1186–1190. 10.1038/nbt.1588 19915550

[7] Berger V, Richter F, Zettlitz K, Unverdorben F, Scheurich P, Herrmann A, et al (2013) An anti-TNFR1 scFv-HSA fusion protein as selective antagonist of TNF action. Protein Eng Des Sel 26: 581–587. 10.1093/protein/gzt044 24006371

[8] Kontos S, Hubbell JA (2012) Drug development: longer-lived proteins. Chem Soc Rev 41: 2686–2695. 10.1039/c2cs15289d 22310725

[9] Roopenian DC, Akilesh S (2007) FcRn: the neonatal Fc receptor comes of age. Nat Rev Immunol 7: 715–725. 17703228 10.1038/nri2155

[10] Ober RJ, Martinez C, Vaccaro C, Zhou J, Ward ES (2004) Visualing the site and dynamics of IgG salvage by the MHC class-I related receptor, FcRn. J immunol 172: 2021–2029. 14764666 10.4049/jimmunol.172.4.2021

[11] Prabhat P, Gan Z, Chao J, Ram S, Vaccaro C, Gibbons S, et al (2007) Elucidation of intracellular recycling pathways leading to exocytosis of the Fc receptor, FcRn, by using multifocal plane microscropy. Proc. Natl. Acad. Sci. USA 104: 5889–5894. 17384151 10.1073/pnas.0700337104 PMC1851587

[12] Hopp J, Hornig N, Zettlitz KA, Schwarz A, Fuss N, Müller D, et al (2010) The effects of affinity and valency of an albumin-binding domain (ABD) on the half-life of a single-chain diabody-ABD fusion protein. Protein Eng Des Sel 23: 827–834 10.1093/protein/gzq058 20817756

[13] Unverdorben F, Färber-Schwarz A, Richter F, Hutt M, Kontermann RE (2012) Half-life extension of a single-chain diabody by fusion to domain B of staphylococcal protein A. Protein Eng Des Sel 25: 81–88 10.1093/protein/gzr061 22238430

[14] Hutt M, Färber-Schwarz A, Unverdorben F, Richter F, Kontermann RE (2012). Plasma half-life extension of small recombinant antibodies by fusion to immunoglobulin-binding domains. J Biol Chem 287: 4462–4469 10.1074/jbc.M111.311522 22147690 PMC3281650

[15] DeLano WL (2000) Convergent Solutions to Binding at a Protein-Protein Interface. Science 287: 1279–1283 10678837 10.1126/science.287.5456.1279

[16] Oganesyan V, Damschroder MM, Cook KE, Li Q, Gao C, Wu H, et al (2014) Structural Insights Into Neonatal Fc Receptor-Based Recycling Mechanisms. J Biol Chem 289: 7812–7824 10.1074/jbc.M113.537563 24469444 PMC3953293

[17] Derrick JP, Wigley DB (1994) The third IgG-binding domain from streptococcal protein G. An analysis by X-ray crystallography of the structure alone and in a complex with Fab. J Mol Biol 243: 906–918. 7966308 10.1006/jmbi.1994.1691

[18] Sauer-Eriksson AE, Kleywegt GJ, Uhlén M, Jones TA (1995) Crystal structure of the C2 fragment of streptococcal protein G in complex with the Fc domain of human IgG. Structure 3: 265–278 7788293 10.1016/s0969-2126(01)00157-5

[19] Müller D, Karle A, Meissburger B, Höfig I, Stork R, Kontermann RE (2007) Improved pharmacokinetics of recombinant bispecific antibody molecules by fusion to human serum albumin. J Biol Chem 282: 12650–12660 17347147 10.1074/jbc.M700820200

[20] Holliger P, Wing M, Pound JD, Bohlen U, Winter G (1997) Retargeting serum immunoglobulin with bispecific diabodies. Nat Biotechnol 15: 632–636 9219264 10.1038/nbt0797-632

[21] Harmsen MM, Van Solt CB, Fijten HP, Van Setten MC (2005) Prolonged in vivo residence time of llama single-domain antibody fragments in pigs by binding to porcine immunoglobulins. Vaccine 23: 4926–4934. 15992972 10.1016/j.vaccine.2005.05.017

[22] Hoffmann E, Konkar A, Dziadek S, Josen HP, Conde-Knape K, Kropp H, et al (2013) PK modulation of haptenylated peptides via non-covalent antibody complexation. J Control Rel 171: 48–56. 10.1016/j.jconrel.2013.06.021 23800420

[23] Sockolosky JT, Kivimäe S, Szoka FC (2014) Fusion of a short peptide that binds immunoglobulin G to a recombinant protein substantially increases its plasma half-life in mice. PLOS One 9: e102566 10.1371/journal.pone.0102566 25057984 PMC4109916

[24] Morell A, Terry WD, Waldmann TA (1970) Metabolic properties of IgG subclasses in man. J Clin Invest 49: 673–680. 5443170 10.1172/JCI106279 PMC322522

[25] Mankarious S, Lee M, Fischer S, Pyun KH, Ochs HD, Oxelius VA, et al (1988) The half-lives of IgG subclasses and specific antibodies in patients with primary immunodeficiency who are receiving intravenously administered immunoglobulin. J Lab Clin Med 112: 634–640. 3183495

[26] Erntell M, Myhre EB, Sjöbring U, Björck L (1988) Streptococcal protein G has affinity for both Fab- and Fc-fragments of human IgG. Mol Immunol 25: 121–126. 3131664 10.1016/0161-5890(88)90059-4

[27] Fahnestock SR (1987) Cloned streptococcal protein G genes. Trends Biotechnol 5: 79–83.

[28] Chaudhury C, Mehnaz S, Robinson JM, Hayton WL, Pearl DK, Roopenian DC, et al (2003) The major histocompatibility complex-related Fc receptor for IgG (FcRn) binds albumin and prolongs its lifespan. J Exp Med 197: 315–322. 12566415 10.1084/jem.20021829 PMC2193842

[29] Spassov VZ, Yan L (2013) pH-selective mutagenesis of protein-protein interfaces: in silico design of therapeutic antibodies with prolonged half-life. Proteins 81: 704–714. 10.1002/prot.24230 23239118 PMC3601434

[30] Watanabe H, Matsumaru H, Ooishi A, Feng Y, Odahara T, Suto K, et al (2009) Optimizing pH response of affinity between protein G and IgG Fc: how electrostatic modulations affect protein-protein interactions. J Biol Chem 284: 12373123–83. 10.1074/jbc.M809236200 PMC2673305 19269963

[31] Baeuerle PA, Reinhardt C (2009) Bispecific T-cell engaging antibodies for cancer therapy. Cancer Res 69: 4941–4944. 10.1158/0008-5472.CAN-09-0547 19509221

[32] Müller D, Kontermann RE (2010) Bispecific antibodies for cancer immunotherapy: current perspectives. Biodrugs 24: 89–98. 10.2165/11530960-000000000-00000 20199124

[33] Finnern R, Pedrollo E, Fisch I, Wieslander J, Marks JD, Lockwood CM, et al (1997) Human autoimmune anti-proteinase 3 scFv from a phage display library. Clin Exp Immunol 107: 269–281 9030863 10.1111/j.1365-2249.1997.254-ce1127.x PMC1904567

[34] Benedict CA, MacKrell AJ, Anderson WF (1997) Determination of the binding affinity of an anti-CD34 single-chain antibody using a novel, flow cytometry-based assay. J Immunol Methods 201: 223–231 9050944 10.1016/s0022-1759(96)00227-x

